# The rhythm of cognition – Effects of an auditory beat on oculomotor control in reading and sequential scanning

**DOI:** 10.16910/jemr.11.2.9

**Published:** 2018-08-20

**Authors:** Elke B. Lange, Aleks Pieczykolan, Hans A. Trukenbrod, Lynn Huestegge

**Affiliations:** Max-Planck-Institute for Empirical Aesthetics, Frankfurt, Germany; University of Würzburg, Germany; University of Potsdam,, Germany

**Keywords:** Reading, visual sequential scanning, background music

## Abstract

Eye-movement behavior is inherently rhythmic. Even without cognitive input, the eyes never rest, as saccades are generated 3 to 4 times per second. Based on an embodied view of cognition, we asked whether mental processing in visual cognitive tasks is also rhythmic in nature by studying the effects of an external auditory beat (rhythmic background music) on saccade generation in exemplary cognitive tasks (reading and sequential scanning). While in applied settings background music has been demonstrated to impair reading comprehension, the effect of musical tempo on eye-movement control during reading or scanning has not been investigated so far. We implemented a tempo manipulation in four steps as well as a silent baseline condition, while participants completed a text reading or a sequential scanning task that differed from each other in terms of underlying cognitive processing requirements. The results revealed that increased tempo of the musical beat sped up fixations in text reading, while the presence (vs. absence) of the auditory stimulus generally reduced overall reading time. In contrast, sequential scanning was unaffected by the auditory pacemaker. These results were supported by additionally applying Bayesian inference statistics. Our study provides evidence against a cognitive load account (i.e., that spare resources during low-demand sequential scanning allow for enhanced processing of the external beat). Instead, the data suggest an interpretation in favor of a modulation of the oculomotor saccade timer by irrelevant background music in cases involving highly automatized oculomotor control routines (here: in text reading).

## Introduction

The human body moves in rhythmic (repetitive) patterns, not only when dancing, but also during walking (step by step), breathing (in and out), and when moving our eyes, the latter being a fundamental constituent of visual cognition. The interplay between rhythm and cognition has mainly been investigated in one direction: how we perceive and produce rhythm, usually in the domain of music psychology (e.g., (31, 54, 78)). However, we here address the more general idea that cognition itself may be rhythmic in nature, especially when associated bodily systems can be regarded as inherently rhythm-based (such as eye movements in the context of visual cognition). The last decades have witnessed growing evidence for an embodied view of cognition (e.g., (8)), a view that also entails the idea that cognitive processes are essentially determined by associated bodily systems (75).

The idea that rhythms can shape cognitive processing has been discussed extensively in the domain of neuroscience. Brain activation contains rhythmic oscillations. Such synchronized activation plays a major role for cognitive processes involved in memory representations and attentional selection (for a review see (14)). Moreover, intrinsic brain rhythms shape visual (5, 24), as well as auditory (28), perception via changes in the excitability of local neuronal ensembles (44). Interestingly, brain rhythms can also be affected by perceptual rhythms. Neural oscillations can be shifted upon perceptual stimulation, an effect called entrainment (44, 43; for a review see (6)). In addition, a single stimulation in one modality (e.g., auditory) can reset the oscillatory phase for processing stimuli from another modality (e.g., visual), demonstrating cross-modal interactions in perception (16, 17, 48). Different from entrainment, auditory-driven phase resets do not need a sequence of auditory stimuli.

There are at least two findings indicating the relevance of brain rhythms for the saccade system. Saccade generation was aligned to visual perceptual oscillations (4, 30). Saccadic reaction times to a visual stimulus onset were reduced by a preceding sound, resulting in crossmodal phase reset (12). That is, attentional dynamics do affect motor behavior.

In the field of visual cognition, classical theories focus on stimulus-dependent processes. However, in recent years the idea of a close coupling of perception and action has transferred to the visual cognition domain, and an “active vision” perspective has been proposed (18), which highlights the role of eye movements as an essential component shaping the visual cognition machinery. Thus, typical everyday visual cognition tasks like reading or searching/scanning for an object have been successfully studied using eye tracking techniques (e.g., 57, 58).

In this context, two diverging views regarding eye movement control have been proposed, namely a “sequential processing” view (e.g., in reading, the EZ-Reader model, see (59, 60)) and a “modulated pulse” view (e.g., in reading, the SWIFT model, see (13); for scene viewing, the CRISP model, see (53); for sequential scanning, see (71)). According to the former, eye movements are driven by an essentially sequential cognitive process aimed at processing stimuli, for example, comprehending words (in the case of reading) or perceiving and categorizing objects (in the case of visual search). Corresponding models therefore assume that eye movements are triggered by a certain stage/level of cognitive stimulus processing (e.g., whether a certain word promises to be decoded successfully, see (60)). Thus, eye movements are triggered only when a certain level of stimulus processing has been reached (“direct control”, (51)).

However, sequential processing models do not adequately capture one crucial aspect of the eye movement system, namely the fact that the eyes “never rest”, but always move. In particular, saccades are continually generated every 250 to 300 ms (57), regardless of any cognitive processing demands. This rhythmic behavior is fundamentally different from other effector systems (e.g., those involving arms, feet, vocal utterances etc.), which usually rest when no movement is required. This special characteristic of the eyes is captured more convincingly in models assuming an autonomous pulse that triggers eye movements (sometimes referred to as “indirect control”, see (32)). Here, cognitive control is regarded as a potential modulator of this pulse (“mixed control”, see (25, 71)). For instance, the pulse is assumed to be slowed down when a difficult word needs to be processed (see (77, 13)). More specifically, prolonged fixation durations are assumed to occur either due to a decreased rate at which the saccade timer accumulates activation until a “move” threshold is reached, or due to a cancellation of an ongoing saccade program (13, 53). There is evidence for “mixed control” in a variety of tasks, e.g. reading (61, 62), scene viewing (27), and visual search (45). The “mixed control” account is also supported by the observation of two distinct saccade populations, of which only one is affected by task settings (52). Whether processing facilitation can also yield a *decrease* of fixation duration is currently under debate (26, 73). Taken together, this “modulated pulse” view of eye movement control seems especially suited to capture the rhythmic nature of eye movement control, which – when taking the embodiment perspective of cognition into account – in turn should shape (visual) cognition. The “modulated pulse” view is also highly compatible with the neuropsychological evidence discussed above: a saccade timer might relate to ongoing brain oscillations (e.g., (4, 12, 30)).

Based on the assumption of rhythmic cognition, we were looking for a method to test the idea of a pulse underlying both visual cognition and oculomotor action in a straightforward but simple way. Based on previous findings of inter-sensory crosstalk and cross-modal attention (e.g., (67)), we reasoned that one promising way to manipulate the putative pulse of visual cognition would be to utilize an external (auditory) pacemaker, that is, a very simple musical stimulus introducing a rhythmic auditory beat. We assumed that rhythmic patterns on the irrelevant (auditory) channel should modulate the pulse in the task-relevant visual processing channel connected to the generation of eye movements. For example, in “modulated pulse” models of saccade generation, the saccade timer is characterized by a random walk to the threshold of saccade initiation. The tempo of musical beats might affect the transition rate of this random walk and alter fixation durations accordingly.

Interestingly, previous studies in the domain of music psychology have already used research designs in which music was presented in addition to, for example, a relevant reading or search/scanning task, but with a completely different research focus, namely to address the question of whether background music (and which type of music) affects performance in the primary task (e.g., scanning: (10, 37); reading: (2, 22, 68)). For example, literature on reading performance showed impaired comprehension in conjunction with task-irrelevant background music (2, 26, 38, 41, 68; but see (42), for a beneficial effect). It has been assumed that processing music requires cognitive resources which conflict with the activation of working memory representations (e.g., (65)). This might also relate to the finding that background music increased fixation durations during scene viewing (66; but see (21)). In line with this argument, background music also impairs performance in a wide range of memory tasks (7). Interestingly, faster music impaired reading comprehension even more than slower music (68). This effect might be due to higher information load with faster music, because more musical events per time unit must be processed. Alternatively, arousal might contribute to the results. Faster music is associated with higher perceived arousal, and high arousal music might impair reading comprehension (11), as well as memory performance (7). There is one study showing faster reading with fast, compared to slow, background music, using a stop-watch to measure paragraph reading on mobile computers in a cafeteria setting (40). This study indicates that indeed eye-movement control might be affected by the tempo of background music.

Corresponding studies using visual scanning tasks have provided mixed results so far as well. Whereas instrumental and lyrical music can speed up performance when presented simultaneously with a scanning task (10), participants took longer to complete a visual search task after exposure to slow versus fast music (37). Taken together, these previous studies on the effects of background music on reading and visual search yield evidence for the possibility of inter-modal (auditory on visual) crosstalk.

In the present study, we focused on two types of visual cognition tasks which are relevant for everyday behavior: (a) text reading, and (b) sequential scanning. Those tasks differ in several aspects. For example, cognitive processing load during reading for comprehension is relatively high, since it involves many memory-based and linguistic processing demands (from syntax to semantics) to extract the meaning of the text (e.g., (33, 34, 35)). In contrast, cognitive processing load (in terms of memory-based and linguistic processing) is particularly low in our utilized sequential scanning task, since the decision of which item should be processed next is predetermined in a simple, task-inherent manner. Specifically, the search array consisted of Landolt-like rings, and the side of the opening of each ring indicated the direction of which object to scan next (“follow the gaps in the Landolt-rings”) until participants found a closed target ring (see (70)).

The two tasks differ also in a second aspect: the underlying oculomotor (instead of cognitive) control demands. Eye-movement control during reading is known to be a highly-learned process characterized by largely autonomous scanning strategies, as indicated by studies showing similar oculomotor patterns when normal text is exchanged by z-strings which lack any semantic content (29, 72). In contrast, selection of the next saccade target in our sequential scanning task is not highly learned and automatized. Instead, it rather depends on moment-to-moment decision-making that is based on meticulous visual attention processing involving detection of the gap location within the stimulus in order to program the appropriate next saccade (70)). Taken together, the two tasks thus differ on two levels of control demands, namely high-level cognitive demands (text reading more demanding than sequential scanning), and lower-level oculomotor demands (sequential scanning more demanding than highly trained and automatized text reading). While, during reading, the ongoing goal of the reader is text comprehension, during scanning, participants should mainly intend to determine the direction of the following saccade.

Our general hypothesis was as follows: if visual cognition is inherently rhythmic in nature (as assumed on the basis of “modulated pulse” accounts of eye movement control in reading and search), it should be possible to influence these processes by employing an external auditory beat of varying tempo. This influence on processing rhythms should become observable in terms of corresponding shifts in temporal eye movement parameters (i.e., a faster beat should yield shorter temporal oculomotor parameters), which in turn reflect temporal characteristics of the underlying cognitive processes.

Based on this general hypothesis, we reasoned that two outcomes are conceivable, depending on how the external pulse is processed by participants:

(a) processing of the auditory beat might consume central (comparatively high-level) processing resources. Then, adding this auditory stimulus might lead to a resource conflict resulting in general slowing of the reading or scanning task. However, the scanning task has a greater chance to be modulated by the auditory beat. Since text reading for comprehension is assumed to be more cognitively demanding than sequential scanning, the auditory beat should be more effective in the latter, because central resources in reading are consumed by the primary task (reading comprehension) and thus no resources are available for processing of the irrelevant auditory stimulus. Additionally, if the auditory beat influences oculomotor control through a more high-level cognitive processing route, one should expect to see effects especially in those oculomotor parameters that are known to be determined by more high-level, cognitive processing. For example, in reading, the central cognitively relevant unit is the word, that is, cognitive oculomotor control (in terms of the decision “where” and “when” to go next) is basically word centered (or object-centered in the case of visual object search, see (58)). Therefore, the assumption of object-based processing predicts effects on gaze durations or total reading times (which are determined by cognitive decisions based on successful word/object decoding), rather than on basic fixation durations. We will refer to this reasoning as the *high-level cognitive load account*.

(b) Processing of the auditory beat might operate on a lower (less cognitive) control level more specifically devoted to basic oculomotor control. On such a basic oculomotor control level, we reasoned that text reading relies on largely automatized oculomotor control routines (i.e., there is relatively low oculomotor control demand), whereas the present sequential scanning task is considerably less trained and associated with oculomotor control decisions from stimulus to stimulus. This greater demand on oculomotor control decisions might prevent any influence of the auditory beat in the sequential scanning task. Instead, oculomotor control during text reading should be affected. Given that this presumable crosstalk operates on a relatively low level of basic oculomotor control, one would expect more basic oculomotor control parameters to be affected (i.e., basic fixation durations instead of gaze durations). This is also plausible since the above cited “modulated pulse” models of oculomotor control are devoted to explaining the control of basic fixation durations. We will refer to this possibility as the *oculomotor control load account*.

There is one important caveat regarding the predictions for temporal parameters that needs to be considered. Previous literature on text reading and sequential scanning has already provided a nearly exhaustive picture of relevant variables determining oculomotor parameters, which together explain a remarkable portion of oculomotor processing variability (e.g., (58), for the case of reading). As a consequence, there is little room left for remaining variables (including external pacemakers) to affect these parameters. Thus, even though we consider the present hypotheses as highly relevant on a theoretical level (as outlined above), it is clear from the start that any potential effects should be very small (i.e., in the range of milliseconds). To make the observation of such small effects more likely, we decided to have participants read through not just one, but many text passages (and to scan through several displays, respectively) in order to maximize the reliability of individual performance estimates. Additionally, we utilized not just two but four different tempi for the auditory beat to further minimize the probability of observing a Type I error, i.e. a false positive result.

In order to implement a more natural experimental situation, we did not use a basic metronome as an external auditory stimulus, but instead composed a melodic-rhythmic pattern (resembling trance-like music with a non-ambiguous, continuous pulse). In this way, our study is also open to interpretation in the context of more applied research questions (i.e., regarding the influence of background music and its tempo on reading and scanning performance, see above for a brief literature review).

The tempi differed strongly, that is between 80 to 140 beats per minutes (bpm), which translates to inter-beat-intervals of 750 to 429 ms or beat frequencies of 1.33 to 2.33 Hz (when regarding the quarter notes as beat throughout, see Table 1). However, fixation durations for silent reading are usually about 225 ms and for scene viewing about 330 ms (see (57)), which translates to frequencies of 3–4 Hz. Importantly, then, the pulse of saccade generation and the beats of our stimuli were on very different time scales. Nevertheless, we expected some (albeit small) modulation effects. Note that our study does not touch on entrainment effects in the narrow sense, which in this case would be reflected in saccades locking onto the beat. First, the range of tempi makes beat entrainment unlikely. Eye movement behavior will unlikely be forced to slow down towards a rate of 1.33 to 2.33 Hz, as given by the quarter beat. However, entrainment to eighths or sixteenths is thinkable but would require a rather complex processing of the simple stimuli. Second, the auditory beats presented in our study were not synchronized in any way to the eye movement recording, rendering a direct relational entrainment analysis impossible. Instead, we simply compared temporal eye movement parameters (e.g., fixation durations) across four different auditory beat conditions to uncover a systematic effect of an external pulse.

**Table 1.** Features of the four tempi applied in the current study.

	Inter-Beat-Interval (ms)			Beat Frequency (Hz)		
Beat (bpm)	Eighth	Quarter	Halves	Eighth	Quarter	Halves
80	375	750	1,500	2.66	1.33	0.67
100	300	600	1,200	3.33	1.67	0.83
120	250	500	1,000	4	2	1
140	214	429	857	4.66	2.33	1.17

*Note.* Inter-beat-intervals and beat frequencies are presented using either the quarter as a reference, which would most likely be perceived as the basis for an intermediate tempo. However, individual beat perception is difficult to predict. When tempo decreases, the perceived beat reference might shift to the eighth (or to the halves for increasing tempo).

## Methods

### Participants

Forty students of the University of Würzburg participated in the experiment (31 female and 9 male) with a mean age of 25 years (*SD =* 3, range = 18–31). All participants reported normal or corrected-to-normal vision and were naïve about the purpose of the experiment. They gave informed consent and received either course credits or were financially compensated (15 €) for their participation. The experimental session took about 2 hours. The study was performed in accordance with the ethical standards described in the Declaration of Helsinki.

### Apparatus

Stimuli were presented on a 21-inch cathode ray monitor (temporal resolution: 100 Hz; spatial resolution: 1024 x 768 pixels). A head and chin rest reduced head movements of the participants. The distance between chin rest and the center of the monitor was 65 cm. Gaze location was registered by measuring the right eye’s pupil using a desktop-mounted infrared reflection system (EyeLink 1000, SR Research, Ontario, Canada) with a temporal resolution of 1,000 Hz. The experiment ran on Windows 7 on a PC. Stimulus presentation and eye movement recording were controlled by Experiment Builder (SR Research, Ontario, Canada).

### Material

**Reading task.** The text material was a subset of a German text corpus created for a different study; a detailed description can be found in (56). The text type was a non-fictional text about the Inuit culture comprising 4,451 words in total. This report was split into 65 passages, with each passage containing six lines of text. Passages were arranged in 5 blocks, with 13 passages per block. Blocks were of similar length (942, 937, 881, 914, 877 words). For each passage, a question regarding the semantic content was prepared, for which the participants had to generate a verbal answer. This procedure ensured proper and attentive reading for comprehension performance. Letter size was 0.44° by 0.24° (height, width) for capital letters and 0.24° by 0.24° for small letters (using an equidistant font). The reading task started with a fixation cross (size: 0.53° by 0.53°) prior to the presentation of each passage.

**Figure 1.**
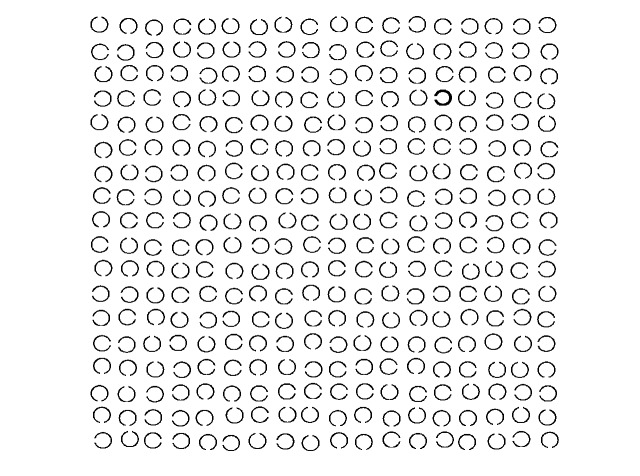
Example of a trial in the sequential scanning task, consisting of 18-by-18 symbols. The start symbol (bold line) is located in column 14, row 4, the end symbol (closed circle) in column 16, row 14 (counting columns and rows from the upper left corner). Participants had to follow a sequence of symbols from the start symbol to the end symbol. A sequence was defined by the openings of Landolt Cs, e.g. from the start symbol: one symbol to the left, followed by one symbol up, one symbol to the left, etc.

**Sequential scanning task.** The task included 50 trials, arranged in 5 blocks with 10 trials each. The number of trials differed from the reading task to adjust for the processing time between tasks. For every trial, a different sequential scanning display was generated beforehand. The visual display consisted of an 18 x 18 grid containing black Landolt Cs_ij_ (i = horizontal position, j = vertical position) on a white background, with a line width of 0.08°, and an opening (gap size: 0.08) at one of the four positions: left, right, top, bottom (see Figure 1 for an example array). Symbols had a diameter of 0.88°. Average horizontal and vertical distance between stimulus elements was 1.32° (measured center to center). The position of Landolt Cs varied horizontally and vertically around the grid centers. This spatial jitter corresponded to one eighth of the size of a Landolt C (± 0.11°). Line thickness of the *start symbol* was doubled. The *end symbol* was defined by a closed ring without a gap. Sequence length of the visual scan path was 50 to 60 symbols in each scanning display.

**Music**. A professional sound engineer created the musical piece using the software Ableton Live 8 (8.0.9) (audio files are available online as Supplementary). The goal was to compose a “musical metronome”, that is a very basic musical stimulus without surprising features. The stimulus comprised three major elements: bass drum, bass-synthesizer, harmony-synthesizer. The bass drum was continuously playing a very simple quarter note pulse, thus ensuring a maximally unambiguous beat and hence tempo perception. The bass- and the harmony-synthesizers played a simple harmonic sequence of tonic, dominant, tonic, subdominant^1^, changing the harmony on the first beat of each bar. The original composition was presented in four different tempi (while keeping all other aspects constant), measured as bpm: 80, 100, 120, 140 bpm. Sound pressure level was kept constant within each session and between participants. It was adjusted to approximately 55-60 dB. A fifth condition without any music served as control condition. Musical stimuli were presented via supra-aural headphones (AKG, model MK 271).

### Procedure

The experimental sessions were conducted individually with one participant at a time. The experimenter was present in order to operate the eye tracking system (i.e., for calibration routines before each experimental block), but seated out of the participant’s sight (to prevent attentional distraction, see (76)). Participants received instructions about the reading/scanning task. They were neither specifically informed or instructed to attend to the music nor to ignore it.

**Reading task.** In the reading task, each block started with a 9-point calibration routine. After successful calibration, the trial began with a fixation cross presented in the upper left quadrant of the screen (located 6.61° from the upper and 3.26° from the left edge of the display). Participants had to fixate it and press the space bar for confirmation. Upon successful fixation, the text was presented with the first letter of the first word at the position of the previous fixation cross. The participants’ task was to read the text and to press the space bar again when they finished reading. This was followed by a comprehension question that appeared centrally on the screen. The experimenter coded manually whether the oral response was correct. To be classified as correct, the response had to convey the meaning, if it did not match the exact wording. Participants initiated the next trial by pressing the space bar. After a delay of 1,000 ms the next trial started.

**Sequential scanning task.** At the beginning of each block, a 13-point calibration routine was performed. We decided for this more precise calibration because of the higher spatial resolution of the display. Each trial began with the presentation of the start symbol. Its position within the 18-by-18 grid was randomized. Participants had to fixate on this symbol and press the space bar for confirmation. Upon successful fixation, the complete 18 x 18 symbol array appeared. Participants were instructed to find the end symbol, i.e., a closed ring, by following a path through the array of Landolt Cs. The path was defined by the openings of symbols. For example, a Landolt C_ij_ with an opening to the left indicated that the next target symbol was located one symbol to the left (position: i-1, j), which in turn indicated the next to-be-fixated symbol by its opening (see Fig. 1; see (69, 70), for an analogue scanning task). When participants reached the *end symbol*, they indicated the search end by pressing the space bar. The next trial started 200 ms after the keypress with a new start symbol.

### Design

Auditory background condition (five levels: music in four different tempi and one silent control) was manipulated as an independent variable within participants in both the reading and the scanning task. The five levels of the auditory condition were blocked, resulting in ten blocks in total. The order of task (reading, scanning) was counterbalanced across participants, with either five blocks reading task followed by five blocks scanning task or vice versa. The auditory background situation was kept constant within one block, but the serial order of the five levels was counterbalanced between participants. The serial order of the trial passages or scanning displays was kept constant. This was necessary for the text reading task, because the text contained a semantically meaningful story and could not be scrambled. The five auditory conditions were chosen to make the following specific comparisons: firstly, the analysis focused on the subset of blocks involving music with four different tempi (effect of tempo), and, secondly, all music conditions were compared with the silent control trials (effect of music presence).

### Data Analysis

One block of one participant was not recorded due to technical failure in the scanning task, so we excluded this participant in the scanning task. We used the automatized sequencing procedure of SR Research (see above) to differentiate fixations and saccades. We defined the space covered by a word or Landolt C and its related blank space as interest areas. The software determines all interest areas in a way that the empty space between symbols (word or Landolt C) is assigned halfway to adjacent areas. For instance, the blank space between words is split into half, with the left half belonging to the prior word and the right half to the succeeding one. We recorded a total amount of 307,106 fixations in the reading task and 202,664 in the scanning task across all participants. Only fixations located in interest areas were further analyzed (exclusion of 0.84% of all fixations in the reading task, 0.02% in the scanning task). We defined fixations with a duration smaller than 75 ms and longer than the mean plus three standard deviations as outliers and excluded them (additional exclusion of 4.53% in reading, 3.28% in scanning). The reading task resulted in 290,617 valid fixations and the scanning task resulted in 195,971 valid fixations. Taking the resulting saccades into account, we recorded 63.56% forward saccades, 19.47% refixations, and 16.97% backward saccades in the reading task. These proportions are in excellent agreement with the reading literature (56, 57). In the scanning task, we primarily observed forward saccades to the next symbol and refixations within a symbol. Proportion of refixations was 40.66% of the data, which is higher than what has been reported before (69, 70), but was likely due to the visually more challenging display (contrary to earlier studies, spatial positions of Landolt-Cs were not located on a perfect regular grid but deviated slightly from this regular arrangement).

For the detected fixations, we then analyzed fixation durations, gaze durations, and total reading times. Gaze durations reflect the duration of the cognitive process underlying the decision of when to move towards the next word. It is a word-based measure and is defined as the summed fixation durations of multiple fixations on one word during first pass reading (i.e., excluding regressions). Our data set spanned 138,923 valid gaze durations (outliers were excluded in a similar manner as for fixation durations). Total reading time is defined as the sum of all fixation durations on one word, including those when a word was fixated multiple times or passages were re-read. As such it is a measure for overall word processing time. Correspondingly, in the scanning task, we summed all fixation durations on a symbol in the 18-by-18 grid. We also analyzed the mean task completion time, which was the time between start of reading a passage or scanning a visual display and the key press of participants signaling the end of the task in each trial.

We deliberately decided to not analyze both tasks within a single statistical model, because the tasks differ in too many respects to allow for a meaningful direct comparison. For example, as outlined in the introduction, word processing in text reading is guided by linguistic control demands and predictive processes based on semantic context, while the scanning task requires moment-to-moment decisions about where to move next in a two-dimensional, highly structured array. In addition, reading results in mostly horizontal saccades, while scanning requires vertical saccades as well, which are known to have a different timing profile than horizontal ones. Finally, making incorrect saccades in the scanning tasks pushes the eyes on the wrong track, e.g., dead ends, whereas the penalty for an incorrect saccade in the reading task is less dramatic. These differences (apart from the general difference between words and Landolt rings) are known to strongly determine oculomotor control, and thus prohibit direct statistical comparisons of both temporal and spatial eye movement parameters across the two tasks. Therefore, we used Bayesian procedures to be able to qualitatively compare result patterns across the two tasks (see below).

Our analysis for each dependent variable was two-fold: first, we incorporated the four music conditions (80, 100, 120, 140 bpm) in a General Linear Model/ANOVA and tested whether a linear trend across increasing tempo conditions emerged. Second, to test for an effect of music presence, we additionally compared performance averaged across all music conditions with the silent control condition.

As a complement to classical ANOVAs, we computed Bayes factors (63, 64). Unlike conventional significance testing, the Bayes factor can be interpreted continuously and quantifies the evidence in favor of one hypothesis over another. Importantly, it also allows us to argue in favor of the null hypothesis. For an interpretation of the magnitude of the Bayes factor, we used Jeffrey’s scale of evidence (46). Bayes factors *BF_10_* larger than 1 generally support hypothesis H_1_ over hypothesis H_0_. Values in the range of 1–3.16, 3.16–10, 10–100, and >100 constitute weak, substantial, strong, and decisive evidence, respectively.^2^ Since Bayes factors are computed as likelihood ratios, Bayes factors BF_10_ smaller than 1 support hypothesis H_0_ over hypothesis H_1_. Accordingly, values in the range 1–0.31, 0.31–0.10, 0.10–0.01, and < .01 constitute weak, substantial, strong, and decisive evidence in support of hypothesis H_0_. In our case we computed Bayes factors to quantify the evidence for a linear effect of tempo and a general effect of music presence, versus the absence of these effects (H_0_). All Bayes factors were computed in R (55) using the *BayesFactor* package (version 0.9.12-2; (50)).

## Results

We first compared fixation durations between tasks in the silent condition. Mean fixation durations in the reading task, *M =* 211 ms (*SD =* 23), matched what is known for typical silent reading (57). In the scanning task, mean fixation durations were somewhat longer, *M =* 308 ms (*SD =* 34). These strong baseline differences, *t*(38) = 17.38, *p* < .001, η^2^ = .888, in addition to the very different underlying task demands discussed above, further strengthen the validity of our decision to separately analyze the reading and the scanning tasks. Results for all dependent variables can be found in Table 2 (reading) and 3 (scanning).

### Reading Task

**Mean fixation duration.** We analyzed all trials, since performance in the text comprehension task was sufficiently high; the mean number of errors ranged between M = 1.45 and 1.90, out of 13 trials for each of the five conditions, and an ANOVA revealed no significant difference in the numbers of errors between the five conditions, *F*(4, 156) = 1.19, *p* = .318, η^2^ = .030. Thus, participants were indeed reading for comprehension as intended.

**Figure 2.**
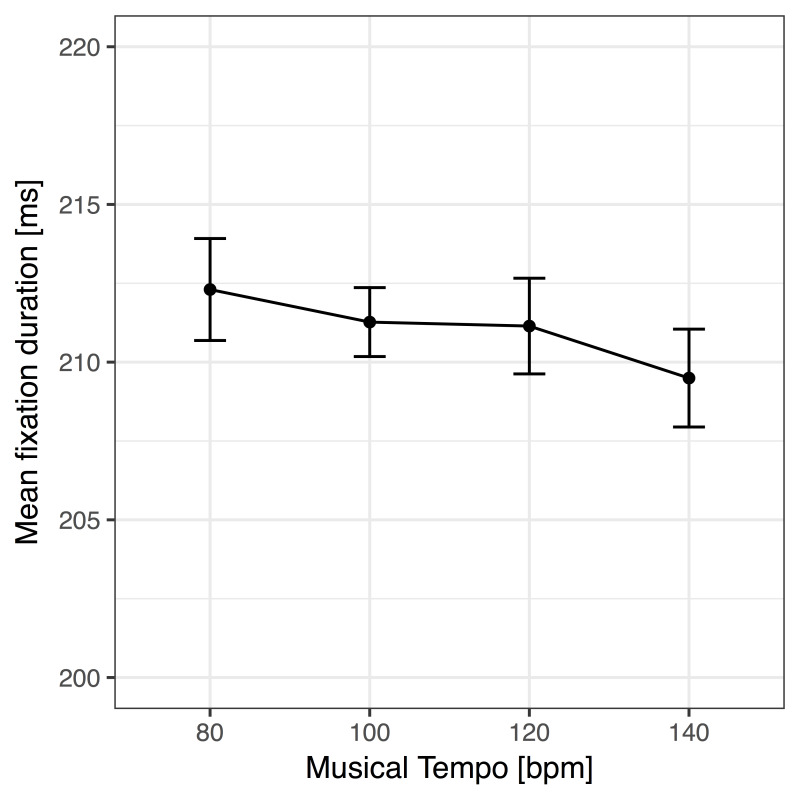
Linear effect of the tempo of the auditory beat on mean fixation duration in the reading task. Error bars show confidence intervals based on (9) and (49).

Importantly, in our ANOVA, including the four music tempo conditions showed a clear linear trend, *F*(1, 39) = 5.61, *p* = .023, η^2^ = .126, which is also depicted in Figure 2. The ANOVA for evaluating the effect of music presence on fixation durations did not yield a significant effect of music versus silent background, *F* < 1, *p* = .904, η^2^ < .001. In line with these results, Bayes factors revealed substantial support for the presence of a linear trend of tempo (*BF_10_* = 4.43), as well as substantial support for the absence of an effect of music presence (*BF_10_* = .23). That is, the mere presence of music did not change mean fixation durations during reading in general, but the tempo of music affected control such that a faster beat reduced fixation durations.

**Table 2.** Summary of ANOVA results and Bayes factors for the reading task.

Analysis	*F*(1, 39)	*p*	η_p_^2^	*BF_10_*
**Mean fixation duration**				
Linear contrast for tempo	*F* = 5.61	.023	.126	4.43^ii^
Effect of music presence	*F* < 1	.904	.000	.23^ii^
**Mean gaze duration**				
Linear contrast for tempo	*F* < 1	.584	.008	.21^ii^
Effect of music presence	*F* < 1	.792	.002	.24^ii^
**Mean total reading time**				
Linear contrast for tempo	*F* < 1	.390	.019	.27^ii^
Effect of music presence	*F* = 7.96	.007	.170	5.38^ii^
**Mean task completion time**				
Linear contrast for tempo	*F* = 1.74	.195	.043	.51^i^
Effect of music presence	*F* = 10.31	.003	.209	12.53^iii^

Note. In our case, Bayes factors BF_10_ > 1 provide evidence for the presence of an effect while Bayes factors BF_10_ < 1 provide evidence for the absence of an effect. Magnitude of the Bayes factor is classified as (i) weak, (ii) substantial, (iii) strong, and (iv) decisive evidence in support of a hypothesis.

**Mean gaze duration.** There was no significant linear trend for tempo, *F* < 1, *p* = .584, η^2^ = .008. Also, music presence did not affect gaze durations, *F* < 1, *p* = .792, η^2^ = .002. Bayes factors showed substantial support for the absence of a linear trend of tempo (*BF_10_* = .21) and for the absence of an effect of music presence (*BF_10_* = .24).

**Mean total reading time.** The results showed a somewhat different pattern than for mean fixation durations. Whereas the linear contrast for tempo was far from significant, *F* < 1, *p* = .390, η^2^ = .019, the main effect of music presence showed reduced total reading times in blocks with music (*M =* 426.68, *SD =* 17.07), in comparison to silent blocks (*M =* 442.66, *SD =* 18.86), *F*(1, 39) = 7.963, *p* = .007, η^2^ = .170. Accordingly, Bayes factors revealed substantial support against an effect of a linear trend of tempo (*BF_10_* = .27) and substantial support for an effect of music presence (*BF_10_* = 5.38).

**Mean task completion time.** There was no significant linear trend for tempo, *F*(1, 39) = 1.74, *p* = .195, η^2^ = .043. Again, and analogous to total reading time for words, the main effect of music presence was significant, *F*(1, 39) = 10.312, *p* = .003, η^2^ = .209. Participants were faster when the reading display was accompanied by music (*M =* 29,505 ms, *SD =* 1,532), in comparison to silence (*M =* 30,702 ms, *SD =* 1,668). While the Bayes factor showed only weak support against the absence of a linear trend (*BF_10_* = .51), we found strong support for the effect of music presence on mean task completion time (*BF_10_* = 12.53).

### Scanning Task

**Figure 3.**
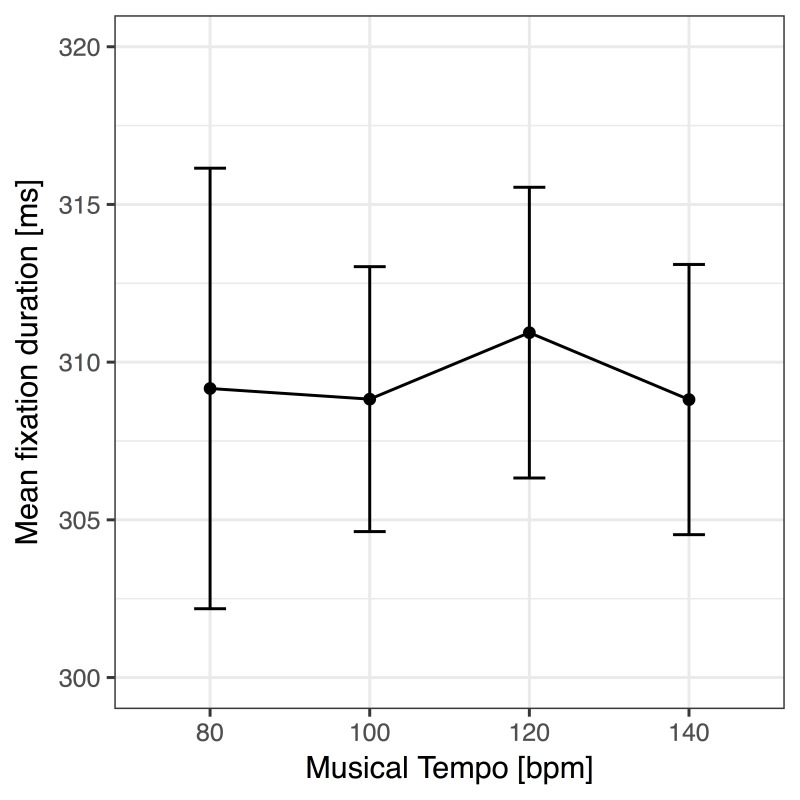
Mean fixation duration as a function of the tempo of the auditory beat. No significant effect was observed. Error bars show confidence intervals based on (9) and (49).

For a summary of the detailed statistics of the scanning task analyses, please see Table 3. All ANOVAs were far from significant, that is, tempo did not have an obvious effect on fixation durations, gaze durations, total scanning times, or task completion times. In addition, musical presence did not show any effect as well. In line with this, Bayes factor analyses provided weak or substantial evidence in support of the null hypotheses (absence of a linear trend of tempo and no effect of music presence; all *BF_10_* < 1). Most importantly, the Bayes factor for mean fixation durations provided substantial evidence against a linear trend (*BF_10_* = .18) (see Figure 3).

**Table 3.** Summary of ANOVA results and Bayes factors for the sequential scanning task.

Analysis	*F*(1, 38)	*p*	η_p_^2^	*BF_10_*
**Mean fixation duration**				
Linear contrast for tempo	*F* < 1	.940	.000	.18^ii^
Effect of music presence	*F* < 1	.703	.004	.25^ii^
**Mean gaze duration**				
Linear contrast for tempo	*F* < 1	.910	.000	.17^ii^
Effect of music presence	*F* < 1	.374	.021	.33^i^
**Mean total scanning time**				
Linear contrast for tempo	*F* < 1	.706	.004	.19^ii^
Effect of music presence	*F* < 1	.546	.010	.27^ii^
**Mean task completion time**				
Linear contrast for tempo	*F* < 1	.363	.022	.32^i^
Effect of music presence	*F* = 2.44	.127	.060	.64^i^

Note. See Table 1.

## Discussion

The present study addresses the general question of whether visual cognition is inherently rhythmic in nature by measuring the potential influence of an external auditory beat, with varying tempo, on temporal eye movement parameters. Specifically, we reasoned that a faster beat should yield shorter temporal oculomotor parameters, which in turn should reflect temporal characteristics of the underlying cognitive processes. We measured eye-movements in two exemplary visual cognition tasks: text reading and sequential scanning. These tasks were either completed in silence or with an auditory beat (simple, electronic music) at four different tempi.

The most important result is that the tempo of the beat significantly affected basic fixation durations in the reading task. Higher musical tempo resulted in shorter mean fixation durations. While the effect was notably small, several aspects strengthen the reliability of the effect. First, it should be noted that we already expected any effect to be small, since eye movements in reading are known to be strongly determined by automatized routines and linguistic processing, leaving only small room for further external determinants. Second, the effect consists of a highly systematic (monotonous) pattern in the expected direction across all four tempo conditions. The implementation of four (instead of only two) tempo conditions makes it unlikely that such an effect represents a random false positive. Finally, the effect was successfully replicated in an independent parallel study involving comparable demands ((47); see below for further details). On the backdrop of our theoretical reasoning outlined in the introduction, this central result is of high theoretical significance.

Specifically, the fact that the Bayes factor analyses provided substantial evidence against an influence of the auditory beat on oculomotor control (mean fixation durations) in sequential scanning but substantial evidence for a reliable effect during text reading can be regarded as evidence for an *oculomotor control load account* and against a *higher-level* *cognitive load account* regarding the underlying level of processing for the auditory stimulus. While oculomotor control in the present sequential scanning task necessitates moment-by-moment decisions on where to move next, oculomotor control in reading relies on highly automatized motor routines, thus leaving more room (in terms of motor control resources) for the processing of the auditory stimulus. If processing of the beat had occurred on a higher cognitive level, one would have expected a strong influence of the beat tempo in the scanning task, but no substantial influence in reading (a pattern we clearly did not observe).

Our present interpretation in favor of the *oculomotor control load account* is further corroborated by the observation that gaze durations (as opposed to basic fixation durations) were not affected by beat tempo during reading. This is in line with “modulated pulse” models of temporal oculomotor control (e.g., (13)), which also assume that an autonomous rhythmic timer determines basic fixation durations to a large degree. Most likely, the external auditory pulse affected the speed of the autonomous saccade timer (rhythmic crosstalk between auditory input and oculomotor output). In contrast, a cognitive account of the effects of the auditory stimulus would have predicted an effect on temporal parameters that reflect more cognitively driven decisions (e.g., gaze durations reflecting the decision of when to fixate the next word, see (57)). However, we did not find any significant effect on gaze durations, which speaks against a *higher-level cognitive load* account of the present data.

Furthermore, the *higher-level cognitive load account* would have predicted a general detrimental effect of the processing demand of the additional auditory stimulus on primary task performance. However, this was not observed in either of the two tasks. While sequential scanning was completely unaffected by the presence of music, total reading times and reading completion times even decreased when the auditory stimulus was present, while text comprehension was not hampered. Several explanations for increased reading speed in the presence of music appear conceivable. For example, music might have increased motivation to complete the task based on positive emotion induction. Or, given its rhythmic nature, the music might have helped participants to stay focused. In addition, the music conditions likely increased arousal in comparison to a silent control, since increased emotional arousal is known to be generally associated with speed (e.g., (3, 15, 23)). Higher arousal might have resulted in the overall effect of speeding up in the mere presence of music; however, it is unclear, why this would affect reading but not scanning.

One difference between tasks is the involvement of articulatory processes in reading (33), as opposed to sequential scanning (where participants might only articulate self-instructions such as “up”/”right” etc.). One might think that when articulating the text silently, participants might match the tempo between articulation (stressed/unstressed syllables) and the auditory beat. In that case, however, the observed effect should have been much more pronounced (similar to an entrainment hypothesis, see Table 1), which renders the assumption of a strategy to match articulation speed with the auditory beat unlikely.

It is important to note that the two tasks, sequential scanning and reading, differed substantially regarding both the underlying processing requirements and the actual oculomotor characteristics, which prohibits any direct statistical comparison between tasks. For example, fixation durations in the scanning task were much longer, and thus the frequency of the oculomotor pulse was actually closer to the chosen frequencies of the auditory beat, but still differed strongly. Future studies on the influence of beat tempo on reading or scanning, particularly when testing entrainment in the sense of typical corresponding studies (see Introduction), should test the effect of pacemaker tempi higher than those utilized in the present study, which were clearly slower than the oculomotor rhythms. Here, we were less interested in such direct entrainment, but rather in the ecologically more prominent situation of background music during visual cognition. Hence the chosen tempi spanned from very slow to rather fast music.

On a more theoretical level, the fact that our results favor an *oculomotor control load account* rather than a *higher-level cognitive load account* also has implications for the underlying cognitive architecture. Previous theoretical frameworks regarding the structural layout of the cognitive system can be divided into models assuming one common central resource for cognitive processing (e.g., (39)) and models assuming a more modular layout (19) involving distinct processing resource pools for the different processing modules (e.g., (74)). A single central module or resource account would have predicted costs associated with additional processing demands, which we did not observe. Thus, our results rather indicate the presence of separate processing modules with rather independent resource pools, one for central, higher-level cognitive processing (linguistic processing, comprehension etc.), and one for more peripheral (and more low-level) oculomotor control, a level on which auditory beat processing appears to operate. However, it appears likely that, despite some degree of modularity, there is still room for inter-modular crosstalk to occur (see (36)), which explains the possibility of an influence of the auditory beat on the oculomotor control system.

Interestingly, the reliability of our main finding is further corroborated by a similarly small but significant effect of musical tempo on fixation durations in free scene viewing as reported recently (47). Note, however, that this study had a somewhat different theoretical focus, comparing the effect of auditory beats between musicians and laymen, as well as between two different musical styles (funk, techno). Only two different tempi were chosen (about 102 and 144 bpm) and task affordances (e.g., differences in involvement of cognitive or oculomotor control processes) were not manipulated. While tempo had an effect similar to the one we observed in our reading task, musical expertise and musical style did not. Despite the differences in theoretical focus, this study therefore nicely confirms our present results and interpretation when assuming that free scene viewing can also rely more on automatized oculomotor scanning routines than the scanning task used in our present study, which necessitates moment-to-moment decisions regarding saccade targets based on the identity of the currently fixated object.

From a more practical viewpoint, our study also speaks to the issue of effects of background music on cognitive task processing. In contrast to other studies (2, 20, 68), we did not find a detrimental effect of music on reading comprehension. Our participants’ comprehension performance was unaffected by the auditory stimulus. In this context, it is important to note that we used very simple musical stimuli, specifically composed for our study. Other studies applied classical or popular music. Such music differs from our stimuli in terms of its complexity and familiarity. For example, music samples taken from top-ten lists might trigger individual memories, associations, and stronger emotional states, potentially conflicting with the reading comprehension task at hand.

Our main finding motivates further research on the relation between auditory rhythms and saccade generation. Even though effects are small and sometimes cannot be observed (see (21), in this special issue of “Music and Eye-Tracking”), they are of high theoretical relevance for understanding crossmodal perception. There are several options for future studies, for example, using experimental designs similar to studies on crossmodal resets in neuroscience (e.g., (12)) and analyzing periodicities for saccade generation (see for example (1)), or using tasks that are suited to uncover different saccade populations (with or without direct control), such as the stimulus-onset delay task (see (52)).

In sum, we demonstrated a theoretically important tempo effect of an external auditory beat on basic eye-movement control in reading. The results are interpreted in favor of modulatory processes that affect the speed parameter of the saccade timer, supporting the assumption of inherently rhythmic underlying processing in exemplary visual control tasks. The present results add to the growing evidence for an embodied view of cognition (e.g., (8)), a view that also entails the idea that cognitive processes are essentially determined by associated bodily systems (75). Thus, tasks requiring rhythmic oculomotor behavior appear to rely on corresponding rhythmic processing strategies, as evidenced by the influence of the task-irrelevant, external pacemaker tempo.

## Ethics and Conflict of Interest

The author(s) declare(s) that the contents of the article are in agreement with the ethics described in http://biblio.unibe.ch/portale/elibrary/BOP/jemr/ethics.html and that there is no conflict of interest regarding the publication of this paper.

## Acknowledgements

We thank Lena Plückebaum and Ruth Julier for data collection. Special thanks to Maria Heuring for her support with the data preprocessing. Special thanks as well to Fabian Greb for generating the musical stimulus. This research did not receive any specific grant from funding agencies in the public, commercial, or not-for-profit sectors. Correspondence concerning this article should be addressed to Elke.Lange@aesthetics.mpg.de

## Footnotes

In music theory, the tonic represents the fundamental key of a musical piece. The dominant and subdominant are the fourth and fifth degree of a diatonic scale. Together, this sequence represents the very simple harmonic progression of a conventional tonal piece of music, e.g. a pop song.

Note that exact ranges correspond to 10^0^–10^1/2^, 10^1/2^–10^1^, 10^1^–10^2^, and >10^2^.
